# Supplementary material for: Label-free nanoscale optical metrology on myelinated axons in vivo
Source: Nat Commun. 2017 Nov 28;8:1832. doi: 10.1038/s41467-017-01979-2 (PMC5705720; doi:10.1038/s41467-017-01979-2)
Supplement: Supplementary file 1 — Supplementary Information [file 41467_2017_1979_MOESM1_ESM.pdf]

Supplementary Information

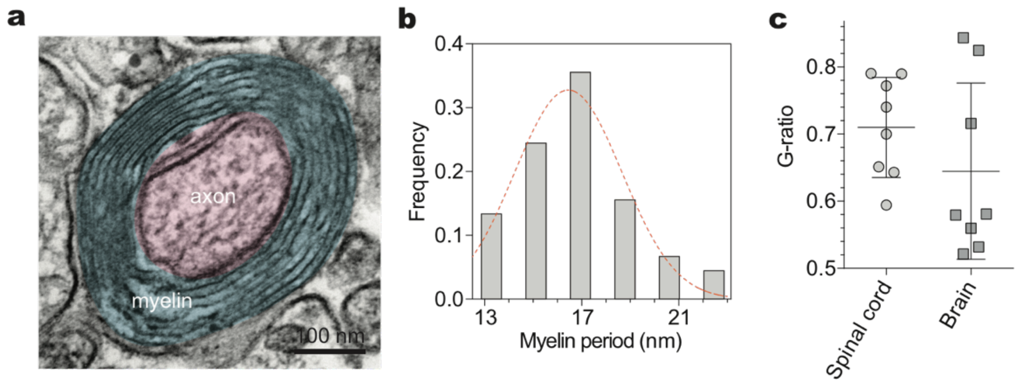

Supplementary Figure 1 | Structural characterization by electron microscopy. a, Transmission electron micrograph of a murine myelinated axon. Pink and cyan pseudo-colors demarcate axon and myelin, respectively. **b**, Histogram of the myelin period with Gaussian fit (n = 45 axons). **c**, Quantification of G-ratio, the ratio of the axonal diameter to the total outer diameter, in the spinal cord and the brain cortex (n = 8 for each group).

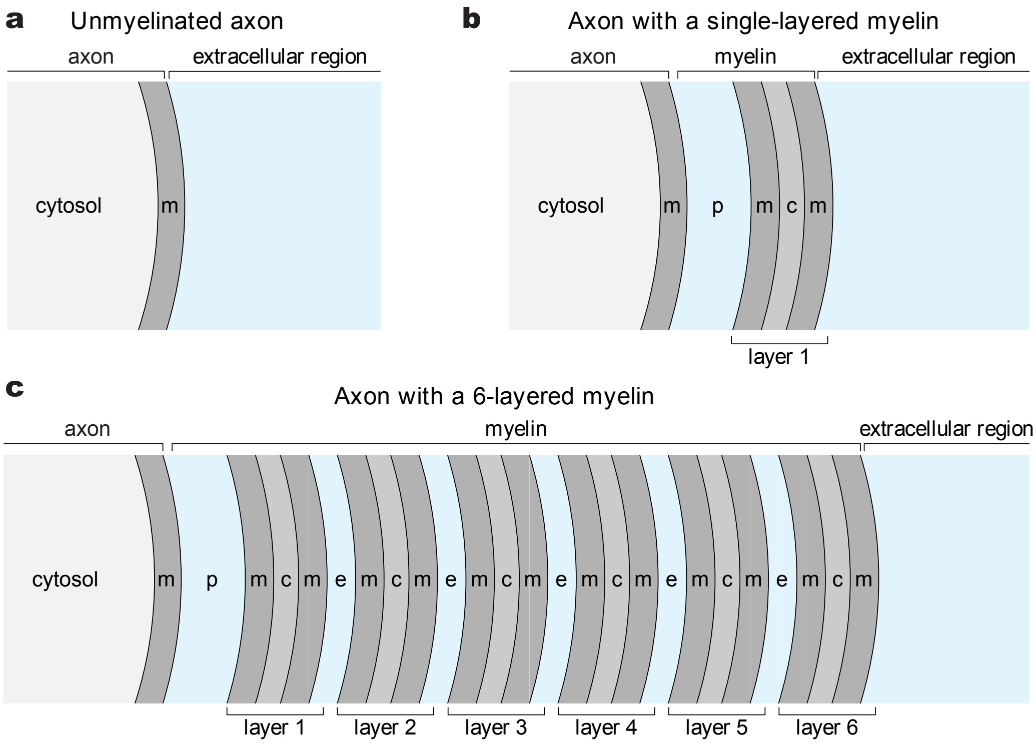

Supplementary Figure 2 | Schematic nanostructure of the axon. a, Unmyelinated axon. **b**, Axon with a single myelin layer. **c**, Axon with 6 myelin layers. m, membrane. p, periaxonal layer. c, cytosol. e, extracellular layer.

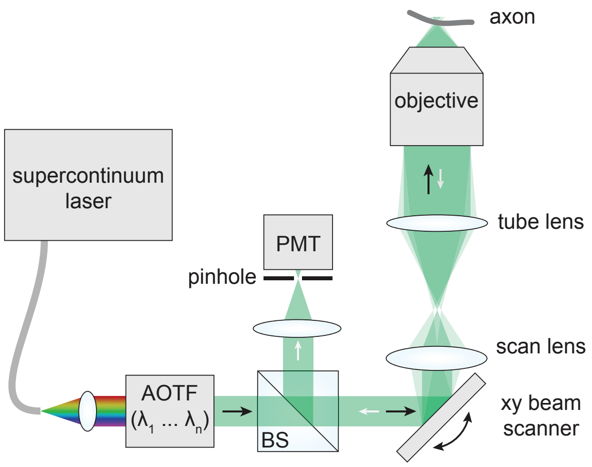

Supplementary Figure 3 | Optic setup for hyperspectral interferometric microscopy. AOTF, acousto-optic tunable filter. BS, beam splitter. PMT, photomultiplier tube.

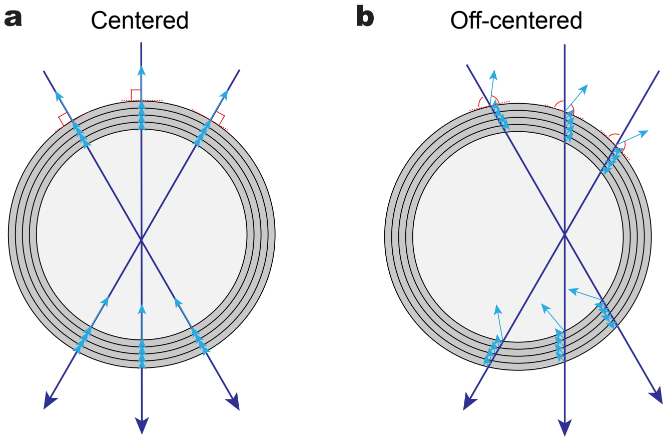

Supplementary Figure 4 | Reflection geometry. a, On-centered: input beam is focused at the geometric center of the axon. Reflected beams (blue) at the interfaces follow the input trajectories and thus detected through a confocal pinhole. **a**, Off-centered: input beam is focused out of the geometric center. Reflected beams (blue) at the interfaces is tilted to the input trajectories and thus rejected by a confocal pinhole.

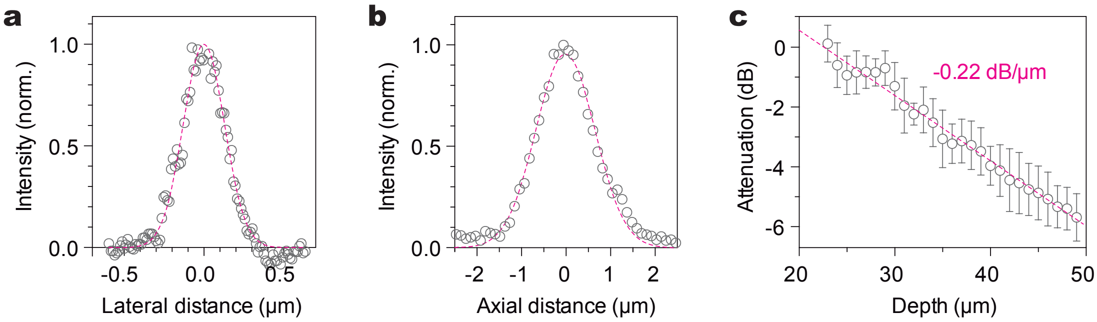

Supplementary Figure 5 | Optical characterization. a-b, Spatial resolution. Normalized visible reflectance from a myelinated axon in the brain cortex are plotted along the lateral (a) and axial (b) axes. Dotted lines in magenta show Gaussian fit to the experimental data (R^2 > 0.97). FWHM (full-width-half-maxium) is 305±10 nm for lateral axis and 1.56±0.07 µm for axial axis. **c**, Depth of penetration. Attenuation of visible reflectance is plotted over depth from the dura mater. Dotted lines in magenta show linear fit.

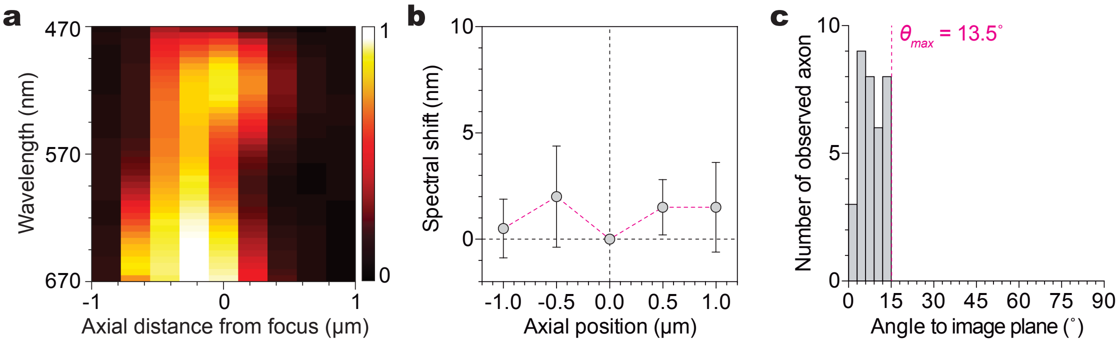

Supplementary Figure 6 | Depth and angle dependency. a, Reflectance spectra measured at the indicated axial distance from the focus. **b**, Quantification of spectral shift (n = 6). **c**, Angular limit of spectral reflectometry. Angle of the axons to image plane is quantified by trigonometry in image stack (total 34 axons). Highest observed angle (θ_{max}) was 13.5°.

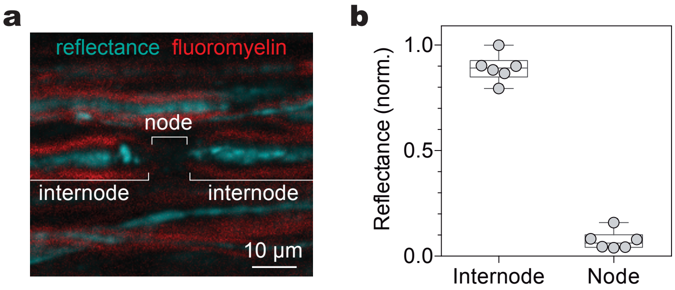

Supplementary Figure 7 | Myelin enhances visible reflectance. a, Spinal nerve imaged by reflectance (cyan) and flouromyelin fluorescence (red). At the node (unmyelinated), both fluoromyelin fluorescence and reflectance are nearly absent. **b**, Quantification of visible reflectance (470–670 nm) signal at the internode and node (n = 6 axons).

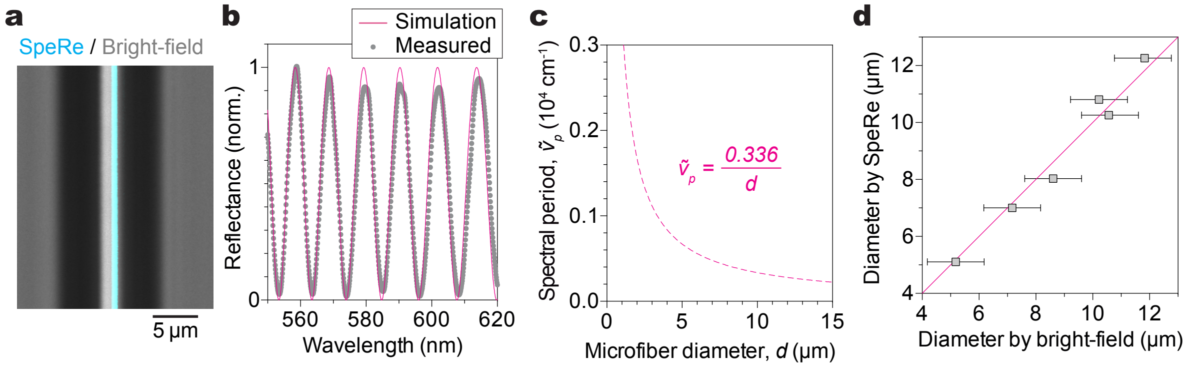

Supplementary Figure 8 | Applicability to polymer microfibers. a, Polymethly methacrylate (PMMA) microfibers imaged by SpeRe (Cyan) and bright-field microscopy (grey). **b**, Representative reflectance spectra in (a). **c**, Theoretical relationship between diameter and wavenumber periodicity ($\tilde{\nu}_p$) acquired by simulation. **d**, Comparison between SpeRe and bright-field measurements (R^2 = 0.97). Error bar indicates diffraction-limited precision of bright-field data.

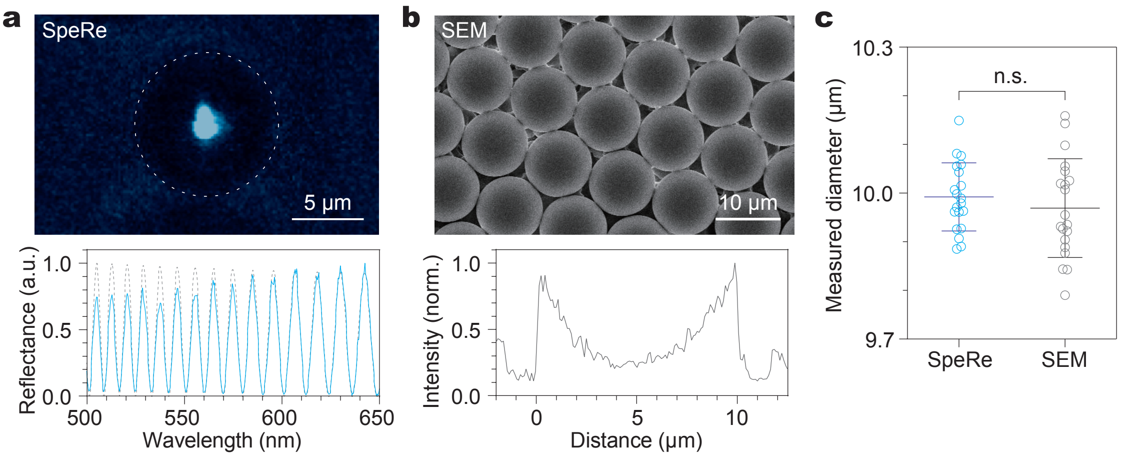

Supplementary Figure 9 | Precision of SpeRe. a, SpeRe on a monodisperse polystyrene bead with a nominal diameter of 10 μm. **b,** Scanning electron microscopy (SEM) on the monodisperse polystyrene beads. **c,** Comparison of diameters quantified by SpeRe (9.99±0.07, n = 20) and SEM (9.97±0.10, n = 20).

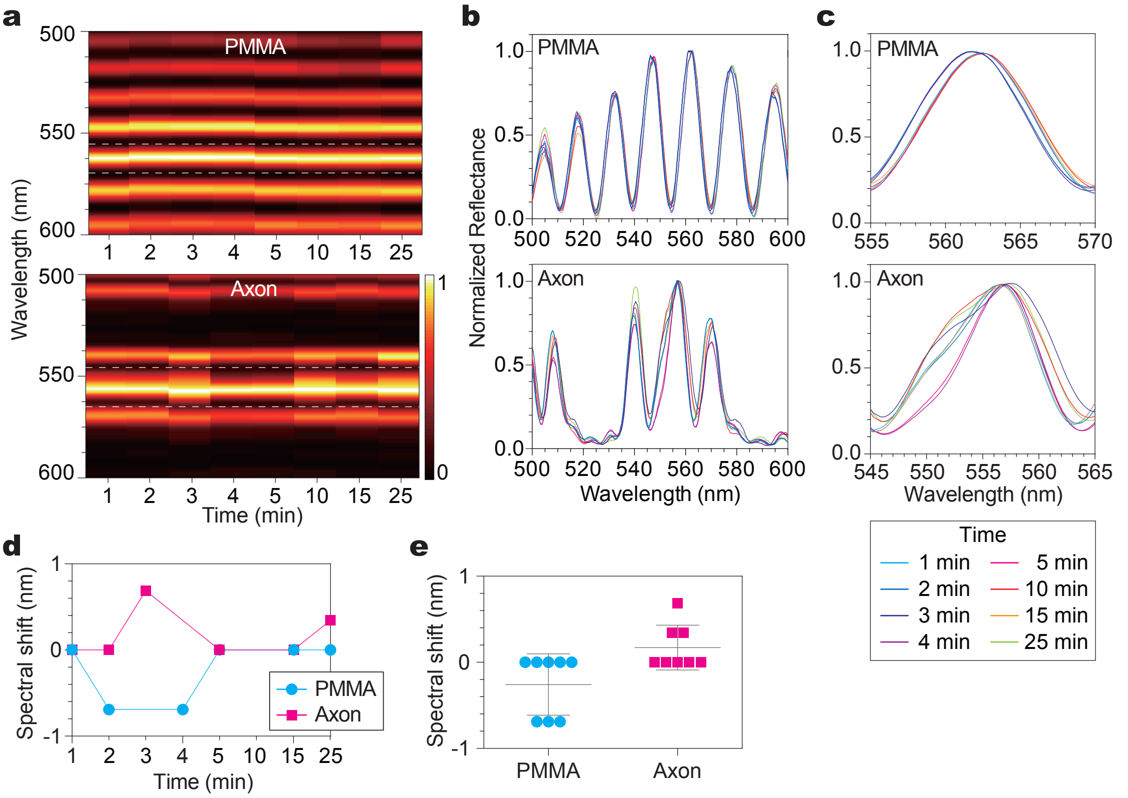

Supplementary Figure 10 | Robustness of SpeRe. a, Repeated SpeRe measurements on a synthetic PMMA fiber and a spinal axon over time (n = 8 each). **b,** Reflectance spectrums over time. **c**, Reflectance spectrums from the dotted wavelength regions in (a). **d-e,** Quantification of spectral shifts (standard deviation: ±0.39 nm for the PMMA fiber, ±0.89 nm for the axon).

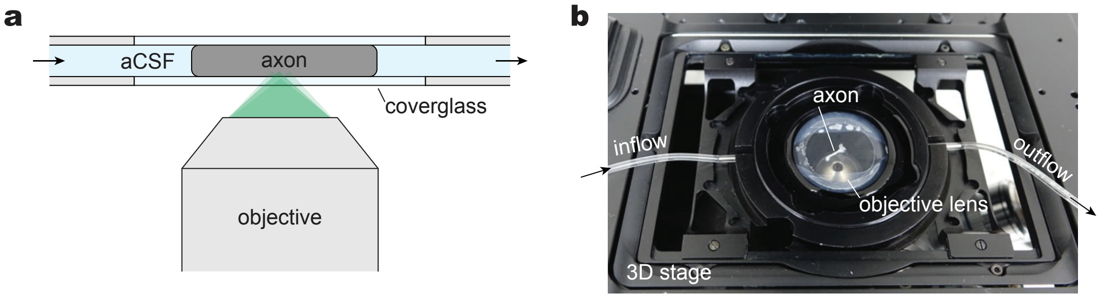

Supplementary Figure 11 | Experimental setup for osmotic challenge. a, Tissue sample is mounted on a fluidic chamber filled with artificial cerebrospinal fluid (aCSF) of varying osmotic strength. **b**, The sample is imaged through an inverted optic setup depicted in the Supplementary Fig. 3.

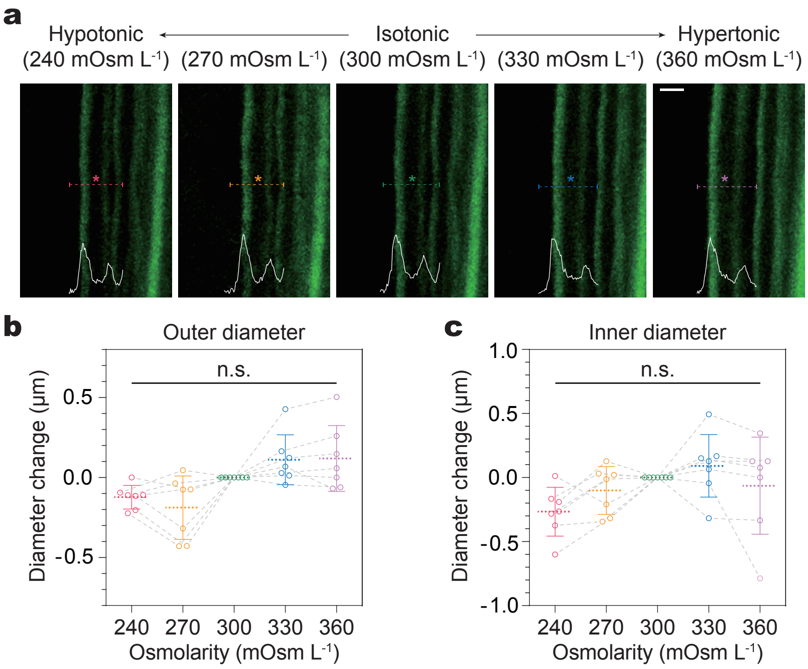

Supplementary Figure 12 | Fluorescence-based quantification of myelin swelling. a, Confocal fluorescence images on flouromyelin-stained myelinated axons in a sliced cortical tissue under osmotic challenge. Scalebar, 3 μm. **b-c**, Quantification of the outer diameter and the inner diameter. Full-width-half-maximum (FWHM) was quantified from the intensity profile (n = 7 axons).

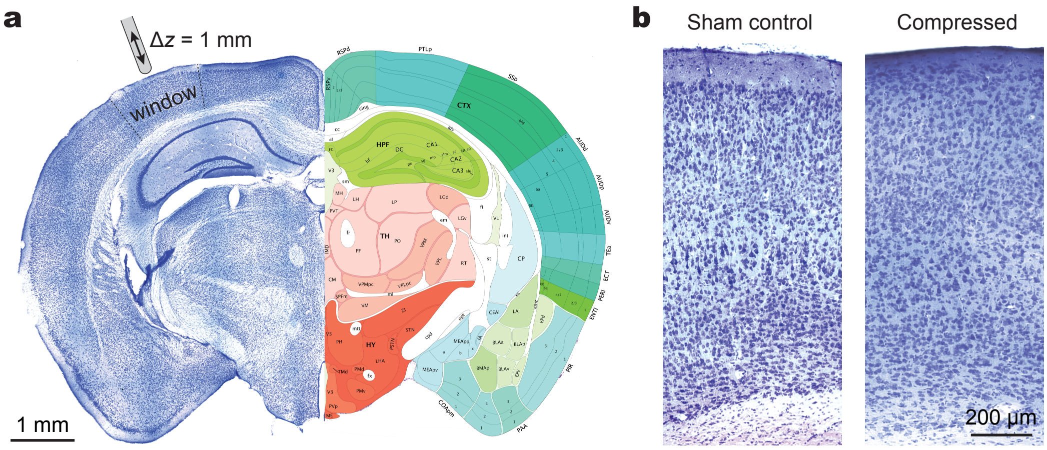

Supplementary Figure 13 | Histological examination of the brain cortex with compressive injury. a, A coronal section of the whole brain with Nissl stain. Dotted lines indicate the region of cranial window. Atlas on the right is adapted from Allen Mouse Brain Atlas (http://mouse.brain-map.org/static/atlas). **b**, Cortical layers of the Nissl stained sections for the sham control (craniotomy only) and for the compressed brain (craniotomy and compressive injury of 1 mm indentation of 30 s).

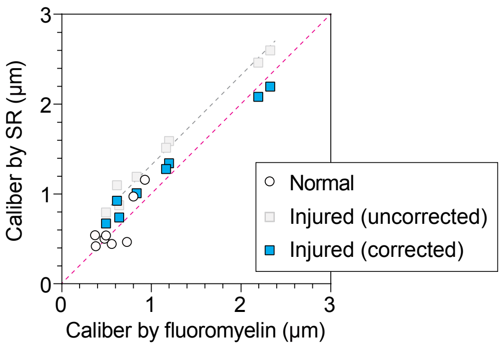

Supplementary Figure 14 | Correction of g-ratio. Estimated axon caliber by spectral reflectometry (SR) and by flouromyelin fluorescence are plotted. When injured axons are assumed to have physiological g-ratio of 0.7 (uncorrected), injured axons (grey square with a dotted fitting line) are notably deviated from the unity line (dotted line in magenta). When g-ratio was corrected according to swelling ratio for each axon (cyan square), axon calibers estimated by SR is well matched with fluoromyelin data (R^2 = 0.93).

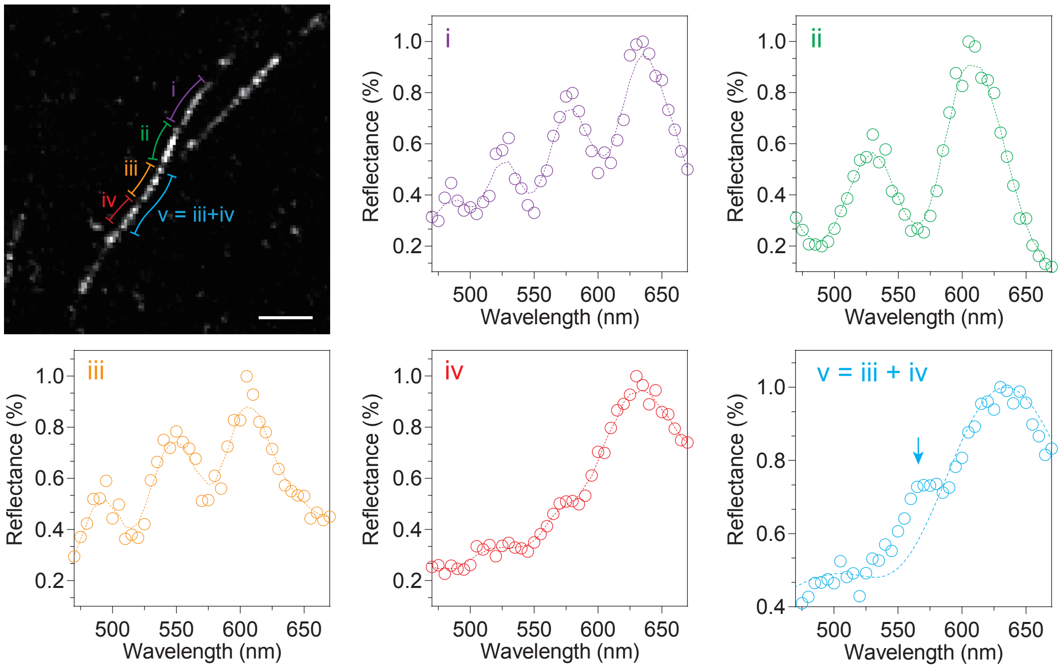

Supplementary Figure 15 | Partial-volume artifact. Reflectance spectrums measured in the indicated axon segments are shown. Note that spectral periodicity (i.e. axon diameter) is heterogeneous along the axon. The partial-volume artifact, such as the bump-like feature (arrow in the lower right panel), can be introduced if the region-of-interest contains heterogeneous structures. Scalebar, 5 μm.

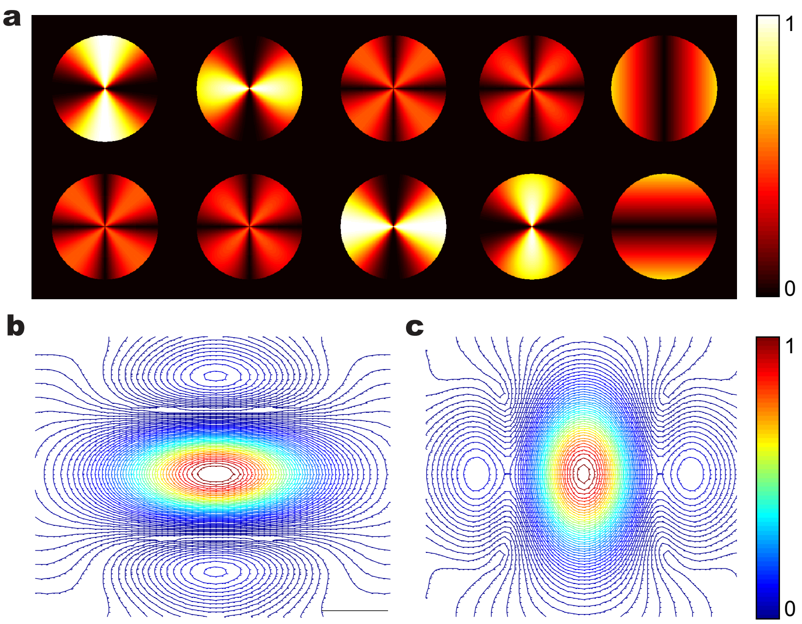

Supplementary Figure 16 | Polarization decomposition of focused wave. a, Amplitude of the polarization decomposition matrix $|D_P|$. Each circular map corresponds to a component of the polarization decomposition matrix, which is delimited by NA of 0.7. Colorbar indicates the amplitude. **b**, Contour plot of the intensity distribution at S polarization on the focal plane. $I_{inc,S}(\mathbf{r})$ scale bar, 300 nm. **c**, Contour plot of the intensity distribution at P polarization on the focal plane. $I_{inc,P}(\mathbf{r})$. Colorbar indicates normalized intensity.

	Thickness	Refractive index
Cytosol for the axon	200 nm – 5 μm	1.36
Lipid membrane	5 nm	1.47 / 1.50
Periaxonal space	12 nm	1.35
Cytosol for the myelin	3 nm	1.47
Extracellular space	5 nm	1.35
Extracellular region	-	1.36

Supplementary Table 1 | Simulation parameters. Detailed methods for determination of the parameters are described in Supplementary Note 1.

Supplementary Note 1. Simulation parameter

1.1 Layer thickness

Structural parameters for myelin substructures are previously reported by electron microscopy and X-ray diffraction studies. Axon diameter is variable from 100 nm up to 5 μm[1]. Axons with their diameter below 200 nm are excluded because they are not myelinated[2]. Thickness of cell membrane is reported to be ~5 nm, including contribution of transmembrane proteins (e.g. phospholipid protein, PLP)[3]. Periaxonal space is reported to be ~12 nm measured by transmission electron microscopy[4]. Myelin cytoplasm is reported to be 3 nm, formed by compaction mediated by myelin basic protein (MBP)[5]. Extracellular layer is set to 5 nm, considering that spatial period of each myelin layer is about 18 nm[6]. Structural parameters used in the simulation are summarized in Supplementary Table 1.

1.2 Number of myelin layer

The g-ratio is defined as the ratio of the inner axonal diameter (*d*) to the total outer diameter (*D*).

$$g - ratio = \frac{d}{D}$$

If the g-ratio is 0.7, the myelin thickness can be expressed as follows.

$$myelin\ thickness = D - d = \frac{d}{0.7} - d = \frac{3}{7}d$$

In our geometric model, the myelin thickness has a discrete value determined by the number of myelin layers, *N* (Supplementary Fig. 2 and Supplementary Table 1).

$$myelin\ thickness = p + N(m + c + m + e) - e$$

where *p*, pericellular space = 12 nm; *m*, membrane = 5 nm; *c*, cytosol = 3 nm; *e*, extracellular space = 5 nm; *N* is an integer. For example, if *d* = 1 μm, myelin thickness is ~428 nm. The number of myelin layer (*N*) is determined as follows.

$$N = round\left\{\frac{428\ nm - p + e}{2m + c + e}\right\} = round\left\{\frac{428\ nm - 12\ nm + 5\ nm}{18\ nm}\right\} = 23\ layers$$

1.3 Refractive index

Refractive indices for each substructure was either obtained from previous literatures or estimated based on reported values. Reported refractive indices were variable among literatures presumably due to difference in sample preparation, measurement condition, and experimental noise but mostly fall within a narrow range. Refractive index of axonal cytosol (n_c) is set to 1.36[7], which is previously reported by phase microscopy technique[8]. This value is also in agreement with cytosolic refractive index of other cell types[7,9]. For cytosolic layer of the myelin, which is direct measurement is not available, we estimated the index based on its composition, by applying Arago-Biot method. Using refractive index and volume fraction for

each amino acid, refractive indice of myelin-associated proteins were estimated, resulting in 1.587 for PLP, 1.588 for MBP and 1.596 for CNP (cyclic nucleotide phosphodiesterase)[10,11]. Volume fraction of MBP on the cytosol is calculated to be ~44% using specific density of 1.38 g/cm^3 and surface concentration of 1.8 mg/m^2. Assuming that cytosol is composed of isosmotic solution (n_S ~ 1.34) with ~44 % of MBP (n ~ 1.59) and with ~2.1 % of CNP (n ~ 1.60), we obtain refractive index of ~1.45 for the cytosol (Eq. 1).

$$n_{cytosol} = (1 - 0.456)n_s + 0.435\, n_{MBP} + 0.021\, n_{CNP} \sim 1.452 \qquad [1]$$

Refractive index of a phospholipid bilayer (n_b) is reported to be 1.46-1.48[3,12,13]. Refractive index of the myelin membrane (n_m) is estimated by weighted sum of a phospholipid bilayer (1.47) and transmembrane proteins, PLP (i.e. Arago-Biot method). In myelin sheath, amount of PLP is about two-fold higher than that of MBP[14]. As MBP constitutes about 44% v/v of the myelin cytoplasm (3 nm in thickness), we estimated that PLP constitutes about 28% v/v of lipid bilayer (10 nm in thickness). With estimated PLP refractive index, n_m is calculated to be 1.50 (Eq. 2).

$$n_m = 0.277\, n_{PLP} + (1 - 0.277)\, n_b \sim 1.502 \qquad [2]$$

Lastly, refractive index for extracellular space (n_e) is estimated. Refractive index of the myelin sheath is consistently reported to be ~1.45[8,15]. Each myelin layer (18 nm in thickness) is composed of 2 layers of membrane (10 nm in thickness), a single layer of cytosol (3 nm in thickness) and extracellular space (5 nm in thickness) as described in Supplementary Fig. 1. As fractional volume for each layer is proportional to each thickness, overall refractive index of the myelin sheath can be written as linear weighted summation (Eq. 3)

$$\frac{10\,nm}{18\,nm} n_m + \frac{3\,nm}{18\,nm} n_c + \frac{5\,nm}{18\,nm} n_e \sim 1.45 \qquad [3]$$

Solving Eq. 3 yields n_e ~ 1.35[16,17]. We used the same index for periaxonal space. Structural parameters for each substructure is summarized in Supplementary Table 1.

Supplementary Note 2. Wave simulation

Numerical simulation based on the theory of electromagnetic waves was performed in order to estimate and predict the spectral reflectance from the myelinated axons of various structures. The distribution of the electric-field reflected from the myelinated axons can be explained by the thin-film matrix theory[18], since myelin layers can be considered as dielectric multilayers. By thin-film matrix theory, matrix components of myelin layers can be defined as,

$$M_j = \begin{bmatrix} \cos\delta_j & i/\eta_j \sin\delta_j \\ i\eta_j \sin\delta_j & \cos\delta_j \end{bmatrix}$$

$$\eta_j = \begin{cases} n_j \cos\theta_j\,, & for\ S-pol \\ n_j / \cos\theta_j\,, & for\ P-pol \end{cases}$$

$$\delta_j = 2\pi n_j \cos\theta_j\, d_j$$

where η_j is optical admittance for S and P polarization at given angle of the ray and δ_j is the

phase delay at the layer. S polarization is perpendicular and P polarization is parallel to the meridional plane, which is defined by plane of incidence. Film matrix M_j is composed of two polarization states since Fresnel reflection coefficient of the medium is dependent on the polarization of the incident light. Then the amplitude reflection coefficient of the myelin is expressed as

$$r_S \; or \; r_P = \frac{B\eta_0 - C}{B\eta_0 + C}$$

$$\begin{pmatrix} B \\ C \end{pmatrix} = \left(\prod_{j=1}^{N} M_j \right) \begin{pmatrix} 1 \\ \eta_0 \end{pmatrix}$$

where, η_0 is the optical admittance at the extracellular region.

Focusing the incident wave into myelin layers at high NA and collecting the reflected light at epi-detection configuration can be described by the wave diffraction theory. The electric field at the position $\mathbf{r}$ on the image plane focused by an objective lens is described by a general scalar form[19].

$$\boldsymbol{E}(\mathbf{r}) = -\frac{if}{\lambda} \iint_{0}^{NA} A(\alpha, \beta) \frac{e^{-i\mathbf{k}\cdot\mathbf{r}}}{\gamma} d\alpha d\beta$$

where $A(\alpha, \beta)$ is the given electric field at the pupil plane of the objective lens, for example, $A(\alpha, \beta) = A_o e^{-(\alpha^2+\beta^2)/NA}$ for a Gaussian beam, f is the focal length, NA is the numerical aperture of the objective lens, α, β, γ are direction cosines which are related to the propagation vector $\mathbf{k}$.

$$\mathbf{k} = (k_x, k_y, k_z) = \frac{2\pi n}{\lambda}(sin\theta cos\varphi, sin\theta sin\varphi, cos\theta) = \frac{2\pi n}{\lambda}(\alpha, \beta, \gamma)$$

$$\alpha^2 + \beta^2 + \gamma^2 = 1$$

where θ, φ are the polar angle and the azimuthal angle along the optical axis. In the scalar form of the electric field, however, variation of polarization according to propagation vector cannot be explained. Polarization decomposition of the incident wave should be considered due to the polarization dependence of multilayers in order to analyze the interaction of the focused light with myelin. To account for local polarization at the incident plane in our high NA optical system (NA of objective lens = 0.7), the vector diffraction theory was employed[20,21]. Thereby, the distribution and polarization of electromagnetic wave focused into multilayered thin-films can be properly described, whereas it cannot be explained in scalar diffraction theory.

The local polarization variation can be described by polarization decomposition factor P_{qlu}, where q is the initial polarization state, l corresponds to the local $\mathrm{S,P}$ coordinate defined on the meridional plane, u corresponds to the global x, y, z coordinate system along the optical axis. The polarization decomposition factor can be derived from the projection of local polarization into global coordinate system as below, which accounts for rotation of the polarization along the propagation vector. P_{xSz}, P_{ySz} are null value since projection of

perpendicular polarization into z axis is zero.

$$D_P = \begin{bmatrix} P_{xSx} & P_{xPx} & P_{xSy} & P_{xPy} & P_{xPz} \\ P_{ySx} & P_{yPx} & P_{ySy} & P_{yPy} & P_{yPz} \end{bmatrix} = \begin{bmatrix} \frac{\beta^2}{1-\gamma^2} & \frac{\gamma\alpha^2}{1-\gamma^2} & \frac{-\alpha\beta}{1-\gamma^2} & \frac{\alpha\beta\gamma}{1-\gamma^2} & -\alpha \\ \frac{-\alpha\beta}{1-\gamma^2} & \frac{\alpha\beta\gamma}{1-\gamma^2} & \frac{\alpha^2}{1-\gamma^2} & \frac{\gamma\beta^2}{1-\gamma^2} & -\beta \end{bmatrix}$$

The figure below indicates amplitude of the polarization decomposition matrix $|D_P|$, which has ten circular maps corresponds to the each polarization decomposition factor. The radius of the circular map is determined by the numerical aperture of the objective lens (Supplementary Fig. 16). Then the electric field incident on the myelin can be expressed as a vector form including polarization decomposition.

$$\boldsymbol{E}_{inc}(\mathbf{r}) = -\sum_{u=x,y,z}\sum_{l=S,P}\sum_{q=x,y}\frac{if}{\lambda}\iint_0^{NA} P_{qlu}(\alpha,\beta)\,A_q(\alpha,\beta)\frac{e^{-i\mathbf{k}\cdot\mathbf{r}}}{\gamma}d\alpha d\beta\cdot\frac{\boldsymbol{u}}{u}$$

The intensity distribution of the focused beam is given by $I_{inc}(\mathbf{r}) = |\boldsymbol{E}_{inc}(\mathbf{r})|^2$. In order to demonstrate the rotation of local polarization, the contour maps of intensity distribution via each local polarization states S and P are indicated in the lower panel of Figure SN, for the given incident Gaussian wave at x polarization.

The electric field reflected from multi-layers of myelin can be derived by multiplying the reflection coefficient into the decomposed electric field at each local polarization state,

$$\boldsymbol{E}_{ref}(\mathbf{r}) = -\sum_{u=x,y,z}\sum_{l=S,P}\sum_{q=x,y}\frac{if}{\lambda}\iint_0^{NA} r_l(\alpha,\beta)\,S(\alpha,\beta)\,P_{qlu}(\alpha,\beta)\,A_q(\alpha,\beta)\frac{e^{-i\mathbf{k}\cdot\mathbf{r}}}{\gamma}d\alpha d\beta\cdot O(\mathbf{r})\frac{\boldsymbol{u}}{u}$$

where $O(\mathbf{r})$ is the overlap function by cylindrical volume of the myelin, $S(\alpha,\beta)$ is the angular weighting function according to structure of myelin. Gaussion function distributed perpendicular to the direction of myelin is suitable for angular weighting function to take into account of the variation of coupling angle of the focused wave due to the concentric cylindrical shape of the myelin. The intensity distribution collected at the epi-confocal geometry is given by

$$I_{ref}(\mathbf{r}) = \left|\boldsymbol{E}_{ref}(\mathbf{r})\otimes\psi_c(\mathbf{r})\right|^2.$$

where $\psi_c(\mathbf{r})$ is the point spread function of the collection system involving confocal detection. Hence, the total reflectance from the myelin at the optical system is given by

$$R = \frac{\iint I_{ref}(\vec{r})dxdy}{\iint I_{inc}(\vec{r})dxdy}.$$

Supplementary References

1. Kasthuri, N. *et al.* Saturated reconstruction of a volume of neocortex. *Cell* **162,** 648–661 (2015).
2. Buzsáki, G. & Mizuseki, K. The log-dynamic brain : how skewed distributions affect network operations. *Nat. Rev. Neurosci.* **15,** 264–278 (2014).
3. Lee, D. W. *et al.* Lipid domains control myelin basic protein adsorption and membrane interactions between model myelin lipid bilayers. *Proc. Natl. Acad. Sci. U. S. A.* **111,** E768-75 (2014).
4. Trapp, B. D. Distribution of the myelin-associated glycoprotein and P0 protein during myelin compaction in quaking mouse peripheral nerve. *J. Cell Biol.* **107,** 675–685 (1988).
5. Chang, K.-J., Redmond, S. A. & Chan, J. R. Remodeling myelination: implications for mechanisms of neural plasticity. *Nat. Neurosci.* **19,** 190–197 (2016).
6. Blaurock, A. E. The spaces between membrane bilayers within PNS myelin as characterized by X-ray diffraction. *Brain Res.* **210,** 383–387 (1981).
7. Curl, C. L. *et al.* Refractive index measurement in viable cells using quantitative phase-amplitude microscopy and confocal microscopy. *Cytom. Part A* **65,** 88–92 (2005).
8. Antonov, I. P. *et al.* Measurement of the radial distribution of the refractive index of the schwann's sheath and the axon of a myelinated nerve fiber in vivo. *J. Appl. Spectrosc.* **39,** 822–824 (1984).
9. Choi, W. *et al.* Tomographic phase microscopy. *Nat. Methods* **4,** 717–9 (2007).
10. Lacourt, A. & Delande, N. Thermo microindentification of amino acids: refractive index. *Microchim. Acta* **52,** 547–560 (1964).
11. McMeekin, T. L., Groves, M. L. & Hipp, N. J. Refractive indices of amino acids, proteins, and related substances. *Adv. Chem.* **44,** 54–66 (1964).
12. Van Manen, H.-J. *et al.* Refractive Index Sensing of Green Fluorescent Proteins in Living Cells Using Fluorescence Lifetime Imaging Microscopy. *Biophys. J.* **94,** L67–L69 (2008).
13. Beuthan, J., Minet, O., Helfmann, J., Herrig, M. & Muller, G. The spatial variation of the refractive index in biological cells. *Phys. Med. Biol.* **41,** 369–382 (1996).
14. Jahn, O., Tenzer, S. & Werner, H. B. Myelin proteomics: molecular anatomy of an insulating sheath. *Mol. Neurobiol.* **40,** 55–72 (2009).
15. Arous, J. Ben *et al.* Single myelin fiber imaging in living rodents without labeling by deep optical coherence microscopy. *J. Biomed. Opt.* **16,** 116012–1160129 (2011).
16. Sun, J., Lee, S. J., Sarntinoranont, M. & Xie, H. Refractive index measurement of

acute rat brain tissue slices using optical coherence tomography. *Opt. Express* **20,** 1084–1095 (2012).

17. Dubowski, J. J. & Tanev, S. *Photon-based nanoscience and nanobiotechnology*. (Springer Science & Business Media, 2007).
18. Macleod, H. A. *Thin-film optical filters*. (CRC press, 2001).
19. Hopkins, H. H. Image-formation with coherent and partially coherent-light. *Photogr. Sci. Eng.* **21,** 114–123 (1977).
20. Flagello, D. G., Milster, T. & Rosenbluth, A. E. Theory of high-NA imaging in homogeneous thin films. *J. Opt. Soc. Am. A* **13,** 53–64 (1996).
21. Richards, B. & Wolf., E. Electromagnetic diffraction in optical systems. II. Structure of the image field in an aplanatic system. *Proc. R. Soc. London A Math. Phys. Eng. Sci.* **253,** 358–379 (1959).
