# Supplementary material for: Label-free nanoscale optical metrology on myelinated axons in vivo
Source: Nat Commun. 2017 Nov 28;8:1832. doi: 10.1038/s41467-017-01979-2 (PMC5705720; doi:10.1038/s41467-017-01979-2)
Supplement: Supplementary file 3 — Description of Additional Supplementary Files [file 41467_2017_1979_MOESM3_ESM.pdf]

File Name: **Supplementary Movie 1**
Description: **SpeRe on a PMMA microfiber.** Reflectance images (cyan) were acquired with spectral scanning from 520 to 600 nm on a synthetic PMMA microfiber. Parallel lines (white) around the reflected signal (cyan) indicate the margin of the fiber. Fiber diameter (10.80 μm) was extracted by analyzing spectral periodicity, as shown in Supplementary Fig. 8.

File Name: **Supplementary Movie 2**
Description: **SpeRe on an axon.** Reflectance images (cyan) were acquired with spectral scanning from 470 to 665 nm on a spinal nerve. In the area demarcated by a rectangle, axon caliber (0.70 μm) and g-ratio (0.69) were extracted by fitting to the simulation data.

File Name: **Supplementary Movie 3**
Description: **3D axon mapping in the mouse cortex.** The spectral image stack is skeletonized, axon diameter for each segment is determined by wavenumber period, and 3D map of the axon is reconstructed. Skeletonization, calculation of axon diameter, and 3D rendering are performed on ImageJ, Matlab, and Imaris, respectively.
